# Analysis of Functional Recovery in Older Adults Discharged to Skilled Nursing Facilities and Then Home

**DOI:** 10.1001/jamanetworkopen.2022.25452

**Published:** 2022-08-25

**Authors:** Sandra Shi, Brianne Olivieri-Mui, Gahee Oh, Ellen McCarthy, Dae Hyun Kim

**Affiliations:** 1 Hinda and Arthur Marcus Institute for Aging Research, Hebrew SeniorLife, Harvard Medical School, Boston, Massachusetts; 2 Northeastern University, Boston, Massachusetts

## Abstract

**Importance:**

Although many older adults are discharged to skilled nursing facilities (SNFs) after hospitalization, rates of patients recovery afterward are unknown.

**Objective:**

To examine postacute functional recovery among older adults.

**Design, Setting, and Participants:**

This cohort study was conducted among older adults treated in SNFs, then at home with home health care (HHC). Participants were a 5% random sample of Medicare fee-for-service beneficiaries discharged to community HHC after SNF stay from 2014 to 2016 with continuous part A and B enrollment in the prior 6 months. Medicare claims data from 2014 to 2016 were used, including inpatient, SNF, hospice, HHC, outpatient, carrier, and durable medical equipment data and Minimum Data Set (MDS) and Outcome Assessment Information Set (OASIS) for SNF and HHC assessments, respectively. Data were analyzed from July 20, 2020, to June 5, 2022.

**Exposures:**

Frailty was measured with a validated claims–based frailty index (CFI) (range, 0-1; higher scores indicate worse frailty) and categorized into not frail (<0.20), mildly frail (0.20-0.29), and moderately to severely frail (≥0.30).

**Main Outcomes and Measures:**

The primary outcome was functional recovery, defined by discharge from HHC with stable or improved ability to perform activities of daily living (ADL). Recovery status was examined at 15, 30, 45, 60, 75, and 90 days after discharge to HHC using OASIS. Covariates were obtained from the MDS admission file at SNF admission, including age, race and ethnicity, cognitive status, functional status, and geographic region.

**Results:**

Among 105 232 beneficiaries (mean [SD] age, 79.1 [10.6] years; 68 637 [65.2%] women; 8951 Black [8.5%], 3109 Hispanic [3.0%], and 88 583 White [84.2%] individuals), 65 796 individuals (62.5%) were discharged from HHC services with improved function over 90 days of follow-up. Among 39 436 beneficiaries not recovered, 19 612 individuals (49.7%) had mild frailty and 15 818 individuals (40.1%) had moderate to severe frailty. While 10 492 of 17 576 beneficiaries who were not frail recovered by 45 days (59.7%), 10 755 of 32 212 individuals with moderate to severe frailty had recovered (33.4%). Overall, frailty was negatively associated with functional recovery after adjustment for demographic characteristics, geographic census regions, and health-related variables, with a hazard ratio for moderate to severe frailty of 0.62 (95% CI, 0.60-0.63) compared with nonfrailty.

**Conclusions and Relevance:**

This study found that recovery after posthospitalization SNF stay was particularly prolonged for individuals with frailty. Functional dependence in activities of daily living remained common among individuals with frailty long after discharge home.

## Key Points

**Question:**

How does recovery vary among patients after receiving home health care in the community after postacute care at skilled nursing facilities?

**Findings:**

In this cohort study of more than 100 000 Medicare beneficiaries who were discharged to skilled nursing facilities and then home after hospitalization, 62.5% had functional recovery within 90 days of home health care admission, and among individuals not recovered, 49.7% had mild frailty and 40.1% had moderate to severe frailty. Frailty was negatively associated with functional recovery, adjusted for health conditions and demographic variables.

**Meaning:**

This study found that many older adults required home health care services for more than 3 months after discharge from skilled nursing facilities, particularly those with underlying frailty.

## Teaser

This cohort study investigates functional recovery by frailty status among older adults discharged from hospitalization to skilled nursing facilities and then home.

## Introduction

Older adults are commonly discharged to skilled nursing facilities (SNFs) after hospitalization owing to hospital-acquired disability and deconditioning.^1,2,3^ While many motives may exist, for many older adults the goal of postacute SNF rehabilitation is ostensibly to recover physical function to enable transition back to the community and eventually full functional recovery.^4,5^ However, recovery is often slow after acute hospitalization. Nearly half of these older adults are not discharged home immediately; instead, many are rehospitalized, die, or move into long-term care.^6,7,8^ Of older adults discharged home to the community who receive home health care (HHC), many still develop new disabilities in activities of daily living (ADL) compared with their premorbid status.^9,10,11^

Older adults with frailty have slower functional recovery owing to diminished physiologic reserve,^12,13^ as found in a smaller prospective cohort study.^3^ Frailty is distinct from physical disability and multimorbidity, instead capturing a state of increased risk for health decline.^14,15^ Thus, even after postacute SNF rehabilitation, individuals may still have high HHC needs.^11^ Although some functional tasks are captured within the SNF setting via standardized forms, such as the Minimum Data Set (MDS), this form is not routinely used in clinical care. Thus, the overall trajectory and time frame to recovery for this high-risk group have not been previously examined, to our knowledge. Often, rehabilitation and HHC are conditional on functional progress in practice; delayed or protracted recovery patterns could be associated with greater likelihood of premature termination of services among patients with increased risk of health decline. Understanding typical recovery is essential for providing informed care to patients and families and ensuring fair and equitable services for older adults with frailty.

Historically, frailty measurement required experienced clinician assessment, but measurement of frailty with electronic health care records and insurance claims data is now possible. This provides exciting opportunities to understand how this important risk factor associated with functional decline is associated with health outcomes at a population level. In the United Kingdom, an electronic frailty index is used to risk stratify older adults, identify those with frailty early, and provide targeted support.^16^ In the United States, a validated claims–based frailty index (CFI) can be calculated from Medicare claims data.^17,18^ The CFI has been used in a wide range of health outcomes studies, including studies on heart failure,^19^ trauma,^20^ and pharmacoepidemiology, such as anticoagulation risk benefit.^21^

Our goal was to examine differences in recovery course after postacute SNF admission by frailty status. We used a CFI to measure baseline frailty status before admission and HHC assessments to examine functional recovery. We hypothesized that frailty, measured by CFI, would be associated with a longer time to recovery among older adults who received HHC after an SNF discharge.

## Methods

The Hebrew SeniorLife Institutional Review Board approved this cohort study and determined that because our data set was deidentified claims data, informed consent was not required. We adhered to the Strengthening the Reporting of Observational Studies in Epidemiology (STROBE) reporting guideline for reporting observational cohort studies.

### Data Sources and Study Population

We used 2014 to 2016 claims data from a 5% sample of Medicare beneficiaries, including inpatient, SNF, hospice, HHC, outpatient, carrier, durable medical equipment (DME) claims, MDS, and Outcome Assessment Information Set (OASIS) data. Participants were eligible for inclusion if they had a short SNF stay between June 14, 2014, and June 30, 2016, defined as a length of stay less than 100 days in MDS, per standard definitions by the Center for Medicare & Medicaid Services.^22^ We included the first observed SNF stay for each beneficiary during this period. Because we were interested in beneficiaries with fee-for-service Medicare who received HHC services after SNF care, we excluded individuals who did not have community discharge per MDS discharge assessment, an OASIS admission assessment within 14 days of MDS discharge assessment, or continuous fee-for-service Medicare enrollment in the 6 months before SNF stay. Thus, patients with Medicare Advantage were excluded, and all participants were presumed to have received sequential care in SNF, followed by HHC.

### Measurements

Demographic factors, including age, race and ethnicity, and geographic region, were obtained from the MDS admission file at the time of SNF admission. Race and ethnicity data are collected as a standard part of the MDS assessment and are queried in a single question, with the following options: American Indian or Alaska Native, Asian, Black or African American, Hispanic or Latino, Native Hawaiian or Pacific Islander, and White. Owing to small sample sizes, the category other was used and includes all groups that were not Black, Hispanic, or White, as well as individuals with missing race data. We included race and ethnicity as a confounder in our adjusted final analyses because race and ethnicity are factors associated with outcomes in postacute care. Cognitive function was calculated from MDS at SNF admission using a standardized cognitive function scale with the following categories: cognitively intact, mildly impaired, moderately impaired, or severely impaired.^23^ Comorbidity burden was measured by the Gagne combined comorbidity index (range, −2 to 26).^24^ ADL were determined from assessments from MDS and OASIS during SNF and HHC stays, respectively. ADL function within SNF was measured using the ADL long-form scale (score range, 0-28; 28 indicates total functional dependence, and 0 indicates no functional dependence).^25^ Additionally, 7 ADL captured consistently across MDS and OASIS were dichotomized into independent or requiring assistance (eTable 1 in the Supplement).

A CFI was calculated based on claims data in the 6 months before SNF admission, defined by the start of care episode in MDS.^17,26^ The CFI uses inpatient, outpatient, SNF, hospice, HHC, outpatient, carrier (fee-for-service claims submitted by professional clinicians, including physicians, physician assistants, and nurse practitioners), and DME claims data. CFI is based on deficit accumulation frailty and thus ranges from 0 to 1, with higher scores corresponding to worse frailty. This outcome was categorized into nonfrailty (≤0.20), mild frailty (0.21-0.29), and moderate to severe frailty (≥0.30), consistent with standard categorization.

We considered 4 possible outcomes after HHC admission: death, rehospitalization, recovery, and nonrecovery. We defined recovery as discharge from HHC with improved ADL function compared with at admission based on a count of 7 ADL that a beneficiary could perform independently as described previously. Nonrecovery was defined as death, hospitalization, or discharge from HHC with worse ADL function compared with at time of HHC admission based on OASIS assessment.

At each prespecified time (15 days, 30 days, 45 days, 60 days, 75 days, and 90 days), we measured outcome statuses by assessing the occurrence of death, hospitalization, or recovery across the overall cohort and stratified by frailty category. With the exception of nonrecovery, once an outcome occurred it was carried forward for future times. For example, if a beneficiary was discharged from HHC services with improved function at 28 days, that individual was considered not recovered at day 15 and recovered from day 30 onward. Only individuals who remained in HHC at the end of our data set (December 31, 2016) were considered administratively censored.

Medicare data sources were linked to the publicly available Certification and Survey Provider Enhanced Reports (CASPER) annual files (2014-2019), which provided information regarding nursing home characteristics, including payer mix and ownership. We classified SNFs based on ownership status (for profit vs government owned or nonprofit), as well as dual eligibility status (Medicare and Medicaid vs Medicare only).

### Statistical Analysis

Demographic characteristics were summarized using mean and SD for continuous variables among the overall cohort and by recovery status. We also calculated median time to recovery by frailty category. Because we were interested in HHC outcomes after an SNF stay, we used Fine-Gray models to estimate hazard ratios (HRs) and 95% CIs between frailty category and functional recovery. Because discharge with recovery cannot be observed once a beneficiary is discharged from HHC for any reason, death, hospitalization, and discharge with worse function were treated as competing risks for functional recovery in these models. We adjusted for demographic variables (age, sex, and race and ethnicity), geographic census region, SNF characteristics, and known confounders to functional recovery, including health-related variables (comorbidities,^24^ cognitive function scale on SNF admission, and ADL function on SNF admission). We then ran models stratified by the top 5 hospital admission diagnoses (eTable 2 in the Supplement). As a sensitivity analysis, we considered all HHC discharges as functional recovery, which did not meaningfully change results. Lastly, we examined recovery status for individual ADL among beneficiaries discharged from HHC upon assessments for SNF admission, SNF discharge, HHC admission, and HHC discharge stratified by baseline frailty status. *P* values were 2-sided and considered significant at *P* < .05. All analyses were performed on Stata statistical software version 15.0 (StataCorp) from July 20, 2020, to June 5, 2022.

## Results

**Figure 1.**
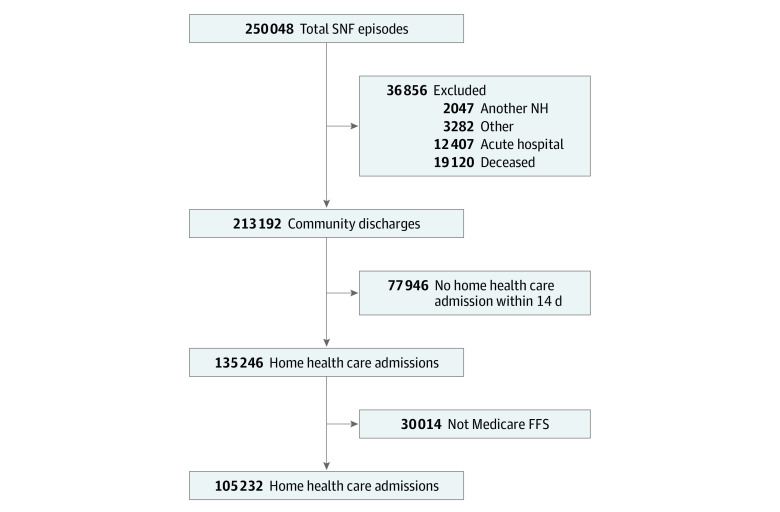
Cohort Flow Diagram FFS indicates fee-for-service Medicare; NH, nursing home; SNF, skilled nursing facility; total SNF episodes, the first episodes of care captured in the Minimum Data Set between July 1, 2014, and June 30, 2016, with a length of stay of 1 day or more and 100 days or less, per Center for Medicare & Medicaid services guidelines for determining short stay. Other exclusions include individuals discharged to psychiatric hospitals, inpatient rehabilitation facilities, hospice, and long-term chronic hospitals.

There were 105 232 beneficiaries included (mean [SD] age, 79.1 [10.6] years; 68 637 [65.2%] women; and 8951 Black [8.5%], 3109 Hispanic [3.0%], and 88 583 White [84.2%] individuals) (Table 1). Among 213 192 beneficiaries discharged from postacute SNF care, 135 246 individuals (63.4%) were admitted to HHC services within 14 days (Figure 1). Overall, 17 576 individuals (16.7%) were not frail, 55 444 individuals (52.7%) were mildly frail, and 32 212 individuals (30.6%) were moderately to severely frail. The mean (SD) number of comorbidities was 5.2 (3.4) comorbidities, and the mean (SD) number of independent ADL on SNF admission was 1.3 (1.3) ADL. Overall, the mean (SD) length of stay in SNF was 29.5 (18.7) days, which was similar for recovered and nonrecovered groups (28.3 days [18.0] days vs 31.6 [19.8] days).

**Table 1.** Demographic Characteristics

Characteristic	Overall (N = 105 232)	Not recovered by 90 d (n = 39 436)^a^	Recovered by 90 d (n = 65 796)^a^
Age, mean (SD), y	79.1 (10.6)	78.8 (11.0)	79.3 (10.3)
Sex			
Women	68 637 (65.2)	24 631 (62.5)	44 006 (66.9)
Men	36 595 (34.8)	14 805 (37.5)	24 631 (33.1)
Race and ethnicity			
Black	8951 (8.5)	4024 (10.2)	4927 (7.5)
Hispanic	3109 (3.0)	1428 (3.6)	1681 (2.6)
White	88 583 (84.2)	32 378 (82.1)	56 205 (85.4)
Other^b^	4589 (4.4)	1606 (4.1)	2983 (4.5)
Region			
Northeast	24 035 (22.8)	8691 (22.0)	15 344 (23.3)
West	27 592 (26.2)	8748 (22.2)	18 844 (28.6)
Midwest	37 694 (35.8)	16 522 (41.9)	21 172 (32.2)
South	15 908 (15.1)	5474 (13.9)	10 434 (15.9)
CFI, mean (SD)	0.27 (0.07)	0.29 (0.07)	0.26 (0.07)
Frailty status			
Not frail (CFI <0.20)	17 576 (16.7)	4006 (10.2)	13 570 (20.6)
Mildly frail (CFI 0.20-0.29)	55 444 (52.7)	19 612 (49.7)	35 832 (54.5)
Moderately to severely frail (CFI ≥0.30)	32 212 (30.6)	15 818 (40.1)	16 394 (24.9)
CFS			
Cognitively intact	73 555 (69.9)	26 058 (66.1)	47 497 (72.2)
Mildly impaired	19 519 (18.6)	8004 (20.3)	11 515 (17.5)
Moderately impaired	10 165 (9.7)	4481 (11.4)	5684 (8.6)
Severely impaired	1002 (1.0)	460 (1.2)	542 (0.8)
Missing	991 (0.9)	433 (1.1)	558 (0.9)
Comorbidity index, mean (SD)^c^	5.2 (3.4)	6.1 (3.4)	4.7 (3.3)
ADL on SNF admission, long-form score, mean (SD)^d^	19.7 (4.4)	20.1 (4.4)	19.5 (4.3)
Independent ADL on SNF admission, mean (SD)	1.3 (1.3)	1.2 (1.3)	1.4 (1.3)
SNF length of stay, mean (SD), d	29.5 (18.7)	31.6 (19.8)	28.2 (18.0)
HHC length of stay, mean (SD), d	48.0 (55.8)	74.0 (86.4)	34.6 (18.3)
For-profit SNF^e^	72 860 (69.4)	28 622 (72.8)	44 238 (67.4)
Dual eligible SNF^e^	93 663 (89.2)	35 514 (90.3)	58 149 (88.6)

Abbreviations: ADL, activities of daily living; CFI, claims-based frailty index; CFS, cognitive function scale; HHC, home health care; SNF, skilled nursing facility.

^a^ Recovery was defined as discharge from HHC services with stable or improved ability to perform ADL.

^b^ Other race or ethnicity includes American Indian or Alaska Native, Asian, and Native Hawaiian or Pacific Islander.

^c^ Comorbidity index was measured with the Gagne Comorbidity Index.

^d^ ADL long-form score on SNF admission ranges from 0 to 28, with higher numbers indicating worse functional status.

^e^ Health care institution data were missing for 277 beneficiaries (0.3%).

**Figure 2.**
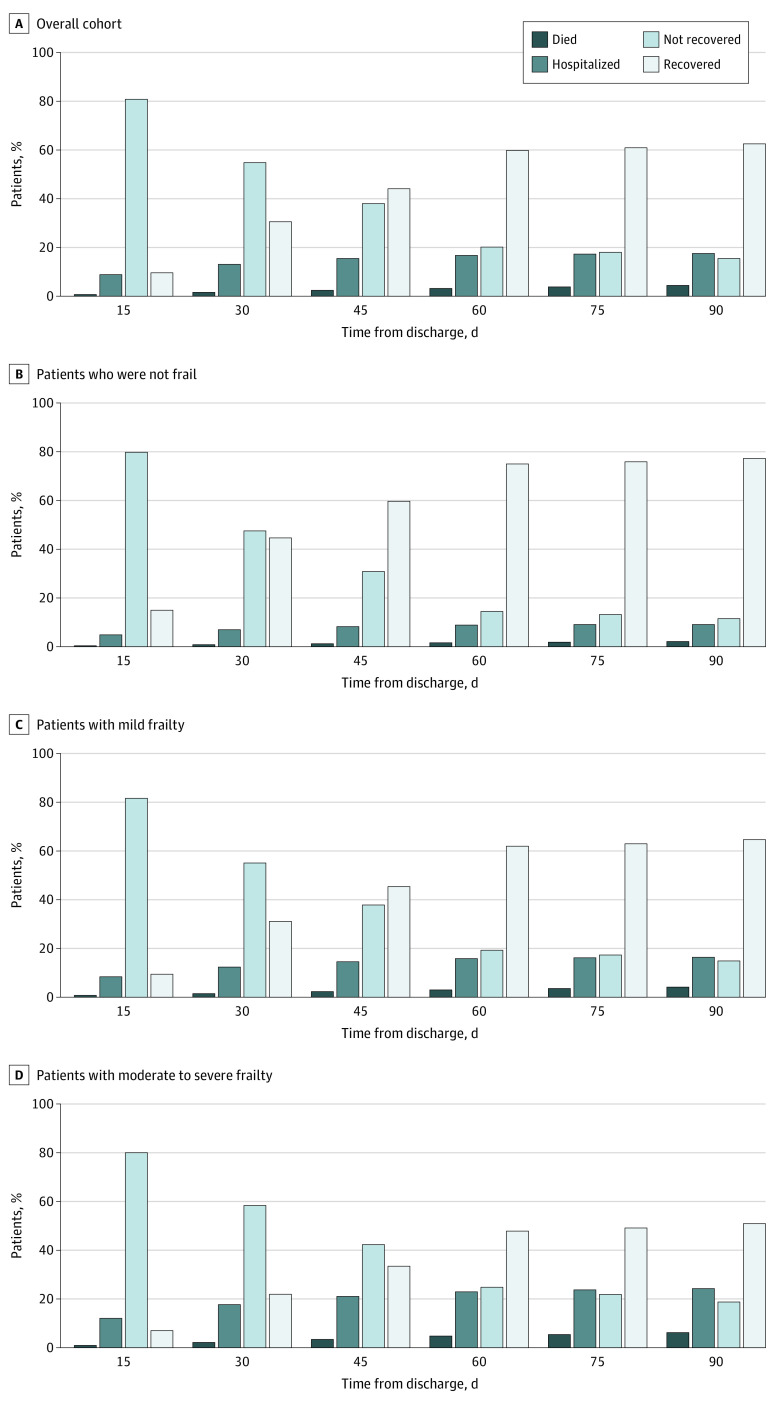
Functional Recovery Over Time Functional recovery at various time points after skilled nursing facility discharge to the community is given overall and by claims-based frailty index. Recovery was defined as discharge from home health care services with stable or improved ability to perform activities of daily living. Frailty defined was categorized by claims-based frailty index score as not frail (<0.20), mildly frail (0.21-0.29), or moderately to severely frail (≥0.30).

During 90 days of follow-up, 65 796 individuals (62.5%) were discharged from HHC services with improved function. Among 39 436 beneficiaries not recovered, 19 612 individuals (49.7%) had mild frailty and 15 818 individuals (40.1%) had moderate to severe frailty. The overall recovery rate plateaued at 60 days (Figure 2). Median (IQR) time to recovery was 33 (21-56) days, 45 (26-82) days, and 82 (32-146) days for adults who were not frail, mildly frail, or moderately to severely frail, respectively. When stratified by frailty status, individuals who were not frail had an earlier recovery, with more than half recovered by 45 days (7847 individuals [44.7%], 10 492 individuals [59.7%], 13 181 individuals [75.0%], and 13 570 individuals [77.2%] recovered at 30, 45, 60, and 90 days, respectively). In contrast, individuals with moderate to severe frailty had a slower recovery, with half of the cohort recovered by 90 days (7058 individuals [21.9%], 10 755 individuals [33.4%], 15 500 individuals [47.8%], and 16 394 individuals [50.9%] recovered at 30, 45, 60, and 90 days respectively) (Figure 2). Over the entire study period, 1774 beneficiaries (1.7%) were discharged from HHC with worsened ADL.

The degree of frailty was negatively associated with recovery (Table 2). In unadjusted models, compared with individuals who were not frail, those with moderate to severe frailty had a recovery HR of 0.46 (95% CI, 0.45-0.47). CFI remained an independent risk factor associated with functional recovery after adjustment for demographic characteristics, geographic census regions, and health-related variables, with an HR of 0.62 (95% CI, 0.60-0.63) among individuals with moderate to severe frailty and 0.78 (95% CI, 0.77-0.80) among those with mild frailty.

**Table 2.** Association of Frailty Status and Patient Characteristics With Functional Recovery

Characteristic	Functional recovery, HR (95% CI)^a^^,^^b^		
	Unadjusted	Adjusted model 1	Adjusted model 2
Frailty status^c^			
Not frail (CFI <0.20)	1 [Reference]	1 [Reference]	1 [Reference]
Mildly frail (CFI 0.20-0.29)	0.68 (0.67-0.70)	0.68 (0.67-0.69)	0.78 (0.77-0.80)
Moderately to severely frailty (CFI≥0.30)	0.46 (0.45-0.47)	0.46 (0.45-0.47)	0.62 (0.60-0.63)
Age, per 1-y increase	NA	1.000 (0.999-1.001)	1.000 (0.999-1.001)
Female sex	NA	1.15 (1.13-1.17)	1.06 (1.04-1.08)
Race and ethnicity			
Black	NA	0.83 (0.80-0.85)	0.87 (0.85-0.90)
Hispanic	NA	0.82 (0.78-0.85)	0.85 (0.82-0.89)
White	NA	1 [Reference]	1 [Reference]
Other	NA	0.99 (0.95-1.02)	1.01 (0.97-1.04)
Region			
East	NA	1 [Reference]	1 [Reference]
West	NA	1.10 (1.07-1.12)	1.11 (1.09-1.14)
Midwest	NA	0.85 (0.84-0.87)	0.85 (0.83-0.86)
South	NA	1.03 (1.01-1.06)	1.03 (1.01-1.06)
CFS^d^	NA	NA	0.94 (0.93-0.95)
Comorbidity index score, per 1 point increase	NA	NA	0.94 (0.94-0.94)
Admission ADL score, per 1 point increase	NA	NA	1.04 (1.04-1.05)
For-profit status	NA	NA	0.91 (0.90-0.93)
Dual eligibility status			
Medicare or Medicaid	NA	NA	1 [Reference]
Medicare only	NA	NA	1.09 (1.06-1.11)

Abbreviations: ADL, activities of daily living; CFI, claims-based frailty index; CFS, cognitive function scale; NA, not applicable.

^a^ Recovery was defined as discharge from home health care services with stable or improved ability to perform ADL.

^b^ Fine and Gray competing risks models are presented unadjusted, adjusted for demographic factors (model 1), and adjusted for demographic factors, cognition, comorbidities, ADL function upon admission to a skilled nursing facility (with higher scores indicating better function), and skilled nursing facility characteristics, including ownership and participation in Medicare or Medicaid (model 2). Death, hospitalization, and discharge with worse function were treated as competing risks for functional recovery.

^c^ Frailty status was categorized into not frail (CFI <0.20), mildly frail (CFI 0.20-0.29), or moderately to severely frail (CFI ≥0.30).

^d^ CFS scores are 1 (cognitively intact), 2 (mildly impaired), 3 (moderately impaired), and 4 (severely impaired). The reference group is individuals with a score of 1, and the outcome is any other score.

Among beneficiaries who were discharged from HHC, the mean (SD) length of time in HHC services was 48.7 (49.2) days, with differences by frailty status (39.2 [37.3], 48.4 [48.3], and 56.6 [57.2] days for individuals who were not frail, mildly frail, and moderately to severely frail, respectively). Among 73 684 beneficiaries discharged from HHC, independence in all ADL was high, ranging from 56 061 individuals (76.1%) for dressing to 72 061 individuals (97.8%) for eating in the total cohort, with the exception of bathing, which 36 322 individuals (49.3% ) were able to perform independently (Table 3). Among 19 066 beneficiaries with moderate to severely frailty who were discharged from HHC services, 6480 individuals (34.0%) were independent in bathing and 12 096 individuals (63.4%) were independent in dressing by the time of discharge from HHC. By comparison, among 14 537 beneficiaries who were not frail and were discharged from HHC services, 9613 individuals (66.1%) and 12 675 individuals (87.2%) were independent in bathing and dressing on discharge, respectively.

**Table 3.** Independence in ADL After Discharge From HHC Services

ADL category	Beneficiaries, No. (%)^a^			
	SNF		HHC	
	Admission	SNF discharge	Admission	Discharge
Total cohort (n = 73 684)				
Grooming	12 729 (17.3)	27 982 (38.1)	43 372 (58.9)	64 862 (88.0)
Dressing	4581 (6.2)	18 525 (25.2)	17 530 (23.8)	56 061 (76.1)
Bathing	3037 (4.1)	10 481 (14.3)	3872 (5.3)	36 322 (49.3)
Toileting	4193 (5.7)	17 752 (24.1)	37 537 (50.9)	63 797 (86.6)
Transfers	4388 (6.0)	19 880 (27.0)	42 004 (57.0)	67 600 (91.7)
Locomotion	9493 (12.9)	27 088 (36.9)	29 887 (40.6)	64 466 (87.5)
Eating	60 573 (82.2)	64 618 (87.8)	70 216 (95.3)	72 061 (97.8)
Not frail (n = 14 537)				
Grooming	3245 (22.3)	6609 (45.6)	10 093 (69.4)	13 815 (95.0)
Dressing	1084 (7.5)	4419 (30.5)	4155 (28.6)	12 675 (87.2)
Bathing	782 (5.4)	2595 (17.9)	1080 (7.4)	9613 (66.1)
Toileting	1010 (6.9)	4327 (29.8)	8595 (59.1)	13 716 (94.4)
Transfers	1007 (6.9)	4703 (32.4)	9021 (62.1)	14 021 (96.5)
Locomotion	2185 (15.0)	6203 (42.8)	6499 (44.7)	13 713 (94.3)
Eating	12 650 (87.0)	13 210 (91.0)	14 215 (97.8)	14 436 (99.3)
Mildly frail (n = 40 081)				
Grooming	6949 (17.3)	15 529 (38.8)	24 021 (59.9)	35 907 (89.6)
Dressing	2515 (6.3)	10 303 (25.8)	9827 (24.5)	31 290 (78.1)
Bathing	1675 (4.2)	5812 (14.6)	2103 (5.2)	20 229 (50.5)
Toileting	2257 (5.6)	9827 (24.5)	20 884 (52.1)	35 347 (88.2)
Transfers	2390 (6.0)	11 005 (27.5)	23 303 (58.1)	37 208 (92.8)
Locomotion	5106 (12.7)	14 890 (37.3)	16 481 (41.1)	35 624 (88.9)
Eating	33 384 (83.3)	35 427 (88.5)	38 421 (95.9)	39 379 (98.2)
Moderately to severely frail (n = 19 066)				
Grooming	2535 (13.3)	5844 (30.7)	9258 (48.6)	15 140 (79.4)
Dressing	982 (5.2)	3803 (20.0)	3548 (18.6)	12 096 (63.4)
Bathing	580 (3.0)	2074 (10.9)	689 (3.6)	6480 (34.0)
Toileting	926 (4.9)	3598 (18.9)	8058 (42.3)	14 734 (77.3)
Transfers	991 (5.2)	4172 (21.9)	9680 (50.8)	16 371 (85.9)
Locomotion	2202 (11.6)	5995 (31.5)	6907 (36.2)	15 129 (79.4)
Eating	14 539 (76.3)	15 981 (83.9)	17 580 (92.2)	18 246 (95.7)

Abbreviations: ADL, activities of daily living; HHC, home health care; SNF, skilled nursing facility.

^a^ Proportion of beneficiaries who could perform a given category of ADL independently at various clinical assessment times.

## Discussion

This cohort study was novel in its examination of functional recovery by frailty status after SNF discharge to home and use of a validated CFI to measure premorbid frailty among older adults admitted to an SNF. We found that recovery after posthospitalization SNF stay was prolonged for individuals with frailty, even after adjusting for comorbidities and SNF admission functional status. Furthermore, ADL dependence remained common among individuals with moderate to severe frailty discharged from HHC, with most such individuals remaining dependent in bathing.

The high rate of discharge from SNF to HHC also raises an important consideration regarding payment structures for sequential stays in postacute care. Presently, Medicare payments are based in specific postacute care settings, with no cost sharing across sites. There have been concerns that payments for HHC would decrease under unified postacute care, and there is no built-in incentive to treat beneficiaries in place and minimize transfers to another postacute care institution.^2^ Using episodes as a unit of care as a form of bundled payment may help to reduce transfer in care while aligning care and cost incentives across the postacute spectrum.^27^

Previous work has focused on factors associated with hospitalization or death in the SNF setting, and few studies have specifically examined outcomes among individuals discharged back into the community. In those studies, receiving HHC services after an SNF was associated with better clinical outcomes,^28^ including lower odds of rehospitalization, SNF readmission, and death.^11,29^ Furthermore, better SNF quality was associated with increased likelihood of HHC referral. Our work highlights that older adults discharged to the community with HHC continued to require care for 3 months or more. Furthermore, many individuals discharged from HHC continued to require assistance in ADL, most notably bathing. Our results suggest that older adults with frailty may need longer SNF stays with more intensive early rehabilitation or different services to enable recovery at home. Future efforts directed at improving transitions in postacute care, particularly in high-risk populations, should consider not only the immediate hospital discharge, but also SNF to HHC transitions.^11,30,31^ Factors associated with reduced hospital admissions during this care transition, such as earlier HHC assessment^32^ and coordination between HHC nursing and outpatient physician follow-up,^33^ should be a focus for potential interventions.

The use of a CFI is particularly informative given that it enables the identification of individuals at increased risk proactively on a health-system level. To our knowledge, this is the first time a CFI was used to assess functional recovery in the postacute setting. Although administrative data collection captures functional information and health status, these data are not readily available to clinicians and frailty is currently not routinely measured in postacute care settings.^34^ With electronic health records enabling automated frailty index calculation,^16,35,36^ a frailty index could be used to flag patients with increased risk at the moment of hospital admission. This may enable, for example, early serious illness conversations or streamlined triage of postacute support services. In fact, the components of routinely collected data could be used to provide frailty measurement at the point of care. Importantly, postacute transitions are often complicated with errors associated with preventable readmissions^37^ and planning that is often limited to short-term medical treatments or plans.^38^ Understanding the expected length of recovery is critical for care coordination and long-term planning for patients and families,^5,28,39^ who may not expect the intensity and duration of support a recovering older adult requires.

It is notable that among beneficiaries who were frail there was a wide variability in time to recovery and recovery status by 90 days. These beneficiaries were less likely to recover than those who were not frail, and ADL dependence was more common by discharge from HHC services. This may reflect the burden of acute stressors and medical conditions, contextual factors, or underlying resilience. This heterogeneity in outcomes highlights a need for better understanding of how the postacute period can optimize functional recovery, independence, and quality of life after hospitalization for older adults. Functional prognostication can be extremely challenging. Although this information is aggregated at a population level, we hope that by providing insights at a broad level we may enable some clinicians to have more informed discussions about long-term functional recovery throughout the postacute spectrum.

While 62.5% of Medicare beneficiaries admitted to HHC after SNF had functional recovery by 90 days, patients who were frail experienced an overall longer duration of HHC services, with less improvement. After 60 days, recovery decreased, possibly reflecting continued high care needs. A 2022 study^40^ among recipients of HHC with cognitive impairment found a high intensity of HHC needs. This is particularly important given that the recently implemented case-mix classification model for HHC reimbursement, the Patient-Driven Groupings Model (PDGM), relies heavily on acute clinical characteristics and does not account for pre-existing vulnerabilities, such as cognitive impairment or frailty. Furthermore, the amount of rehabilitative treatments provided no longer factors into payments, raising concerns that populations with increased risk and a greater degree of functional impairment may receive fewer services.^41,42^ Overall, this model may inadvertently disincentivize the care of older adults who are frail.

The plateau in overall recovery at approximately 60 days was surprising. Recovery was conservatively defined as discharge from HHC with any improvement from admission status; however, this does not necessarily mean that patients achieved maximal benefit given that they may no longer have met Medicare requirements (eg, homebound status) for HHC services. Maintenance of function is extremely important for high-risk populations,^43,44^ and thus absence of improvement does not necessarily mean that the services are unnecessary. HHC services also provide nursing care, including wound care services and medication management. These are critical and inextricably tied within the care provided by HHC agencies. In the context of the newly proposed PDGM model, which shortens payment periods from 60 days to 30 days,^45^ services for beneficiaries who are frail and have delayed recovery may be disproportionately impacted. The shortened time horizon may not accommodate or enable full functional recovery for patients at highest risk. For those on the precipice, preliminary withdrawal of HHC services may tip them toward rehospitalization or institutionalization.

### Limitations

This study has several limitations. The decision process for postacute care planning is complicated with contextual factors, including local variation in practice, SNF bed availability, and patient preferences. To be sure, care should be individualized to the greatest extent possible in clinical care, and we lacked granularity to determine such individualization with claims data alone. Although we used a validated CFI, some diagnoses common to older adults who are frail, including dementia and delirium, are often undercoded; thus, CFI may underestimate frailty in beneficiaries at highest risk. However, our adjusted analyses incorporating cognitive function from MDS still found an association between CFI and functional recovery. We found that 63.4% of individuals discharged from postacute SNF care were admitted to HHC services; these results are generalizable only to Medicare fee-for-service beneficiaries discharged to a community setting from SNF. Additionally, the associations described here predate the COVID-19 pandemic and may have changed with efforts to streamline postacute care processes.

MDS and OASIS are regulatory databases, and their data are not collected specifically for research. However, these data have been approved as reliable and valid tools for population health studies.^46^ Given that we were unable to directly measure prehospitalization function, our baseline for recovery was functional ADL status at HHC admission. Thus, functional recovery in this study may not represent true return to baseline functional status. In a prospective cohort study^47^ examining functional trajectories after hospitalizations, recovery beyond baseline was rare. We acknowledge that discharge with worse function does not preclude future improvement in physical function. Given that our definition of functional recovery was contingent on discharge with improved function, discharge with worse function was considered mutually exclusive from functional recovery. In our sample, discharge with worse function was rare (1.7%), suggesting that most individuals who were not improved continued to require services at home. Thus, defining functional recovery by need for services may allow for a pragmatic and clinically meaningful outcome definition.

## Conclusions

This cohort study found that among Medicare beneficiaries discharged to the community after SNF who received HHC, functional recovery varied by pre-SNF admission frailty status. Our study suggests the utility of CFI in early identification of at-risk populations who experience delayed functional recovery. Further work should examine possible modifiers to this functional trajectory to inform intervention development for this high-risk population.

## Notes

Supplement. **eTable 1.** Operationalized Crosswalk Between Minimum Data Set and Outcome 2 and Assessment Information Set for Activities of Daily Living **eTable 2.** Association Between Frailty by Claims-Based Frail Index and Functional Recovery by Primary Diagnosis for Hospitalization
