# Supplementary material for: Analysis of Functional Recovery in Older Adults Discharged to Skilled Nursing Facilities and Then Home
Source: JAMA Netw Open. 2022 Aug 25;5(8):e2225452. doi: 10.1001/jamanetworkopen.2022.25452 (PMC9412223; doi:10.1001/jamanetworkopen.2022.25452)
Supplement: Supplement. — eTable 1. Operationalized Crosswalk Between Minimum Data Set and Outcome 2 and Assessment Information Set for Activities of Daily Living eTable 2. Association Between Frailty by Claims-Based Frail Index and Functional Recovery by Primary Diagnosis for Hospitalization [file jamanetwopen-e2225452-s001.pdf]

Supplemental Online Content

Shi S, Olivieri-Mui B, Oh G, McCarthy E, Kim DH. Analysis of functional recovery in older adults discharged to skilled nursing facilities and then home. *JAMA Netw Open*. 2022;5(8):e2225452. doi:10.1001/jamanetworkopen.2022.25452

eTable 1. Operationalized Crosswalk Between Minimum Data Set and Outcome 2 and Assessment Information Set for Activities of Daily Living
eTable 2. Association Between Frailty by Claims-Based Frail Index and Functional Recovery by Primary Diagnosis for Hospitalization

This supplemental material has been provided by the authors to give readers additional information about their work.

© 2022 Shi S et al. *JAMA Network Open.*

eTable 1. Operationalized Crosswalk Between Minimum Data Set and Outcome 2 and Assessment Information Set for Activities of Daily Living

	INDEPENDENT	DEPENDENT
**MDS**		
Bed Mobility (G0110A)	0, 1	2, 3, 4, 7, 8
Locomotion on Unit (G0110E)	0, 1	2, 3, 4, 7, 8
Dressing (G0110G)	0, 1	2, 3, 4, 7, 8
Toilet Use (G0110I)	0, 1	2, 3, 4, 7, 8
Personal Hygiene (G0110J)	0, 1	2, 3, 4, 7, 8
Eating (G0110H)	0, 1	2, 3, 4, 7, 8
Bathing (G0120A)	0, 1	2, 3, 4, 8
**OASIS**		
Transferring (M1850)	0, 1	2, 3, 4, 5
Locomotion (M1860)	0, 1, 2, 4	3, 5, 6
Dressing (M1810, M1820)	0, 1	2, 3
Toilet (M1840)	0, 1	2, 3, 4
Toilet Hygiene (M1845)	0, 1	2, 3
Grooming (M1800)	0, 1	2, 3
Eating (M1870)	0, 1	2, 3, 4, 5
Bathing (M1830)	0, 1	2, 3, 4, 5, 6

Note: Each ADL available in both datasets was dichotomized as supervision or better. Final ADL counts used in the manuscript are the sum of total number of independent or supervised ADLs. In OASIS Dressing is broke down into upper and lower body. Notably this is a rough operationalized translation of these differing instruments, which vary in time frame and language. Abbreviations MDS – Minimum Data Set OASIS - Outcome and Assessment Information Set

© 2022 Shi S et al. *JAMA Network Open.*

eTable 2. Association Between Frailty by Claims-Based Frail Index and Functional Recovery by Primary Diagnosis for Hospitalization

**Primary Hospitalization Diagnosis Group**		**Unadjusted Model**	**Adjusted Models**
**Cardiovascular (n=19,642)**	Non-Frail	Reference	Reference
	Mild Frailty	0.76 [0.72 - 0.80]	0.75 [0.71 - 0.79]
	Moderate to Severe Frailty	0.55 [0.52 - 0.58]	0.55 [0.52 - 0.58]
**Trauma and Fracture (n=16,007)**	Non-Frail	Reference	Reference
	Mild Frailty	0.76 [0.73 - 0.79]	0.76 [0.73 - 0.79]
	Moderate to Severe Frailty	0.56 [0.53 - 0.59]	0.56 [0.53 - 0.59]
**Musculoskeletal (n=13,223)**	Non-Frail	Reference	Reference
	Mild Frailty	0.68 [0.66 - 0.71]	0.68 [0.66 - 0.71]
	Moderate to Severe Frailty	0.40 [0.37 - 0.42]	0.40 [0.38 - 0.43]
**Pulmonary (n=8,078)**	Non-Frail	Reference	Reference
	Mild Frailty	0.76 [0.69 - 0.84]	0.76 [0.69 - 0.84]
	Moderate to Severe Frailty	0.52 [0.47 - 0.58]	0.54 [0.49 - 0.60]
**Infection (including sepsis) (n=7,062)**	Non-Frail	Reference	Reference
	Mild Frailty	0.79 [0.71 - 0.87]	0.79 [0.71 - 0.88]
	Moderate to Severe Frailty	0.56 [0.50 - 0.62]	0.57 [0.51 - 0.63]

Note: The top five diagnosis groups for admission are represented above.

Adjusted Models adjusted for age, sex, race/ethnicity status, and geographic region.

© 2022 Shi S et al. *JAMA Network Open.*
